# *“Suddenly we have hope that there is a future”*: two families’ narratives when a child with spinal muscular atrophy receives a new drug

**DOI:** 10.1080/17482631.2021.1904722

**Published:** 2021-03-31

**Authors:** Elin Hjorth, Malin Lövgren, Ulrika Kreicbergs, Thomas Sejersen, Eric Asaba

**Affiliations:** a Department of Health Care Sciences, Palliative Research Centre, Ersta Sköndal Bräcke University College, Stockholm, Sweden; b The Department of Women’s and Children’s Health, Paediatric Oncology and Haematology, Childhood Cancer Research Unit, Karolinska Institutet, Karolinska University Hospital, Stockholm, Sweden; c The Department of Women’s and Children’s Health, Paediatric Neurology, Karolinska Institutet, Karolinska University Hospital, Astrid Lindgren Children’s Hospital, Stockholm, Sweden; d Division of Occupational Therapy, Department of Neurobiology Care Sciences and Society (NVS), Karolinska Institutet, Stockholm, Sweden; e Research, Education, Development, and Innovation Unit, Stockholms Sjukhem Foundation, Stockholm, Sweden; f Occupational Therapy & Occupational Science Research Group, Department of Health Sciences, Lund University, Lund, Sweden

**Keywords:** Spinal muscular atrophy, neuromuscular disease, families, narrative inquiry, hope, resilience

## Abstract

**Purpose:** This study aims to explore negotiations of hope in everyday life for families where a child with spinal muscular atrophy (SMA) has received a new drug treatment.

**Methods:** A narrative design was used, drawing on interviews and participant observations in two families with children with SMA, types 1–2, to situate family experiences of hope in everyday life. Narrative analysis was used on the data.

**Results:** Results are presented as stories, with details about situations and contexts, to illustrate how hope was used by families to reconstruct their own family narratives.

**Conclusions:** Hope was negotiated and struggled with in different ways by different family members, but contributed to each person’s own way of dealing with the disease and outlook for the future.

## Introduction

Spinal muscular atrophy (SMA) is a rare and severe muscular disease affecting young children. Long-term disease among children has been described as a family affair, affecting the entire family (Knecht et al., 2015). Families challenged by long-term disease face adversities in different ways; some can easily find and use resources, while others need extensive support to cope with the disease (Wright & Leahey, 2012).

SMA is genetic and is characterized by gradually increasing muscle weakness. SMA is commonly divided into three levels of severity, SMA types 1–3. Without treatment, a child with the most severe form, SMA type 1, usually dies within the first two years of life due to difficulties in breathing and swallowing. In the absence of treatment, children with SMA type 2 usually have a life expectancy into young adulthood, while life expectancy in SMA type 3 is unaffected (Lunn & Wang, 2008). Until just a few years ago, there was no treatment available affecting the progression of muscle weakness in SMA. All treatment therefore focused on preventing complications from muscle weakness and maintaining quality of life (Arnold et al., 2015). However, recently, promising treatments have become available on the market, which has changed the SMA landscape dramatically. After clinical trials that showed slowed progression of muscle atrophy, improved survival in infants and children, and in some cases even regain of previously lost muscular functions (Al-Zaidy et al., 2019; Finkel et al., 2017; Mercuri et al., 2018), nusinersen (Spinraza) was approved by the US Food and Drug Administration in 2016. In Sweden, nusinersen was approved in December 2017 and is now available to children with SMA fulfiling certain criteria (The New Therapies Council (NT-rådet, 2017). Another therapy, Zolgensma, is a gene replacement therapy administered through a single, intravenous dose that was approved in 2019 by FDA for infants with SMA who are less than two years old.

The advances in medical treatment have changed the situation for families with a child with SMA. For instance, it was recently shown that a non-ambulatory child with SMA type 2 was able to walk as a direct result of nusinersen, something previously not possible for children with SMA type 2 (Darras et al., 2019). Further, if nusinersen treatment is started at a pre-symptomatic stage, ambulation is achieved also in many children otherwise expected to follow the progressive and lethal course of SMA type 1 (Darryl et al., 2019). However, although the therapy is effective and for many children results in functional improvement, some children do not improve (Pane et al., 2018; Pechmann et al., 2018).

Within the context of disease experience, everyday hope is described as essential for finding and maintaining the energy to live. Literature includes reviews on hope in samples of various age groups, diagnoses, and family members. One review of families of children living with chronic disease found that hope was dynamic and that families used different strategies to balance hope (Leite et al., 2019). Hope can be present for parents throughout the disease trajectory and during the palliative phase, expressed as hope for a cure, for meaningful time with their child, and for a pain-free death (Van der Geest et al., 2015). Parents of children with neuromuscular disease have described moving between feelings of hope, avoidance, and living in the present (Erby et al., 2006). Hope can be expressed in different ways with different benefits, from helping parents to absorb the initial crisis to facilitating their adaptation or preparing them for a fatal outcome (Samson et al., 2009). Within paediatric care of children with severe diseases, the approach to hope can differ between parents and professionals. While parents underline their role as bearers of hope and protectors of their children, professionals describe a tension between the emotional aspect of hope and the difficulty of maintaining hope in the face of prognostic data (Reder & Serwint, 2009). Current evidence suggests that it is necessary to be honest about the prognosis, but there is no need to discourage parents from hoping for a cure (Robinson, 2012; Van der Geest et al., 2015). Literature of children’s experience of hope when living with a disease is scarce. However, hope has been suggested to play a unique role in paediatric contexts by being an important factor to increase resilience, reduce levels of anxiety, and improve youths’ health (Griggs & Walker, 2016; Lewis & Kliewer, 1996; Martins et al., 2018).

Although studies involving families with chronic or severe illness emphasize the importance of hope, there is more to explore given the complexity of hope. SMA is particularly interesting, since it has gone from being a life-limiting disease to one that with treatment may be associated with long life. In the SMA context, the hope for a potential cure has suddenly become a reality and families with children who have SMA are now facing hope in new ways. Building on knowledge situated in the intimacy of moments and stories shared by families, the present study epistemologically draws on narratives (Mattingly, 2010) with the aim to explore negotiations of hope in everyday life for families where a child with SMA received a new drug treatment.

## Method

### Design

The study used a qualitative design in which narrative interviews (Anderson & Kirkpatrick, 2016) and participant observations (Atkinson & Hammersley, 2007) were carried out in a naturalistic setting. Narrative inquiry refers here to stories, told and enacted, about human actions gathered through events, and narratives co-constructed throughout the data gathering phase (Mattingly, 1998; Polkinghorne, 1995). Moreover, narrative analysis was used to bring otherwise disparate data together into a coherent whole, and to integrate theory and interpretation in the presentation of results, to situate participant experiences in a socio-cultural context. Narrative inquiry, with an alignment from data gathering to analysis and presentation, allows for abstract concepts to be tested against scenarios tacitly present in everyday life. The results are thus integrated with theory and presented in a storied format.

### Recruitment and data generation

All non-bereaved parents who participated in a previously conducted nationwide Swedish survey (Hjorth et al., 2018; Lövgren et al., 2016a, 2016b) were invited to participate in this study together with their family (six families). In addition, recruitment took place via a public advertisement at the Swedish patient association for SMA (Facebook and website). To participate, the family had to include at least one child between 6 and 19 years diagnosed with SMA type 1 or 2.

Family was defined in keeping with an inclusive view: “the family is who they say they are” (Wright and Leahey, 2012). This meant that the families in this study were invited to decide whom to include in their family constellation. Two families were selected for the present study as they consented to having the first author be present in their homes several times: Alexander’s family, including three children and two parents, and Isaac’s family, including two children, two parents, and a close relative. None of the researchers had a previous relationship with either of the participating families.

**Figure 1.**
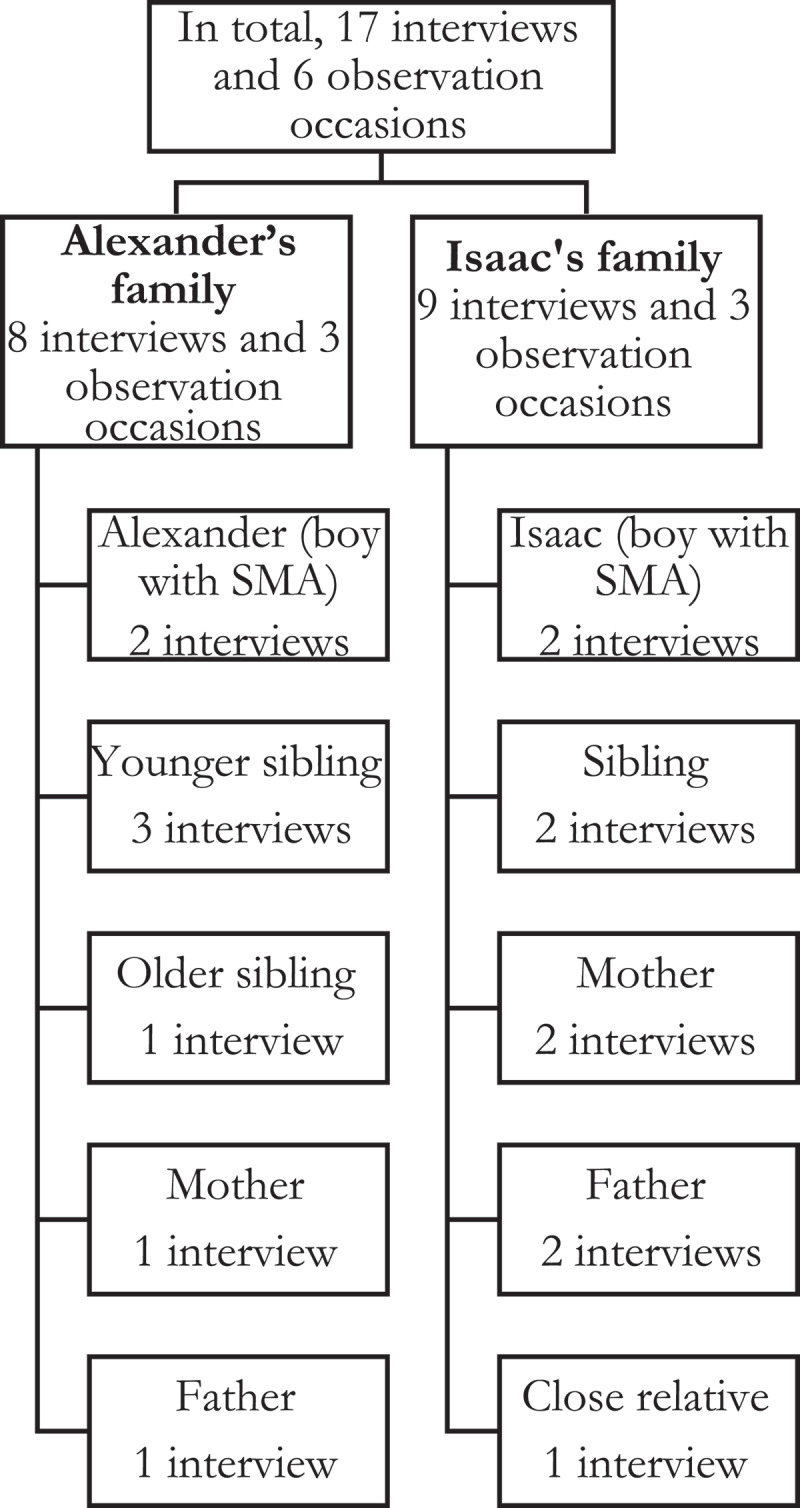
Number of included interviews and observations

Data were generated over a period of 9 months. Six meetings took place, comprising 17 interviews with the different family members and participant observations with field notes (Figure 1). Repeated visits with the families took place, in which participant observations were used to gain a rich understanding of the families’ experiences of living with the disease and negotiating hope (Atkinson & Hammersley, 2007). The first author took the role of a guest in everyday life, meaning that she participated in events to which she was invited, e.g., sitting together on a sofa while the kids were playing, socializing during meals, or attending school events. Shorter field notes, including some quotes, were written during the observations, and more extensive notes, including a variety of elements, were written in direct connection with each visit (Reeves et al., 2008).

Narrative interviews with open questions were conducted in connection with the observations to allow for storied responses and to promote sharing of experiences from the informants’ perspective. Interview questions focused on daily life with the disease, experiences of support from healthcare and society and expectations on treatment. No specific questions were pre-formulated, but areas of interest were identified by the research group before the interviews were conducted. These were inspired by reading relevant literature, from other studies, and by conversations with parents of children with SMA. The participant observations also raised further questions, which were used to follow up any outstanding queries. After each visit with a family, field notes and transcriptions of the interviews were reviewed. The data were discussed within the research group, to deepen understandings of the material between interviews. Things that were somehow unclear, were followed up in coming interview.

The recorded interviews were mainly conducted individually, except those with Alexander and Isaac, as they wanted/needed a parent nearby. The first author conducted all interviews and observations, and continuous reflections and discussions with co-authors took place during the entire data gathering process. The interviews lasted 10–40 minutes with children (average 20 minutes), and 25–70 minutes with adults (average 50 minutes), and were digitally recorded and transcribed verbatim.

### Data analysis

The analysis began with transcription of the first interview, which enabled alignment of follow-up questions in subsequent visits and interviews. Interviews and field notes were analysed with narrative analysis (Josephsson & Alsaker, 2015; Mattingly, 1998), a method using understanding of significant events within narratives, by reconstructing otherwise isolated experiences as a coherent whole. By focusing attention on the wholeness of the narrative, not on common experiences across different people, narrative analysis does not fragment stories and experiences into parts or categories (Creswell & Poth, 2017). Interpretations were shaped by alternating focus on specific parts with focus on the whole (Josephsson & Alsaker, 2015; Lindseth & Norberg, 2004; Mattingly, 1998).

The first author read through all the transcribed interviews and field notes several times and took notes in order to get a deeper understanding of the data. In next phase, significant events that “stood out” in the data were observed and discussed within the research group. In this process, the narratives about hope and treatment of SMA were identified as significant events. The more specific aim for this study was formulated, and data corresponding to the aim were sorted out and re-constructed into narrative vignettes (Creswell & Poth, 2017; Josephsson & Alsaker, 2015). The vignettes were processed and shortened with a focus on the purpose of the study. In parallel with this process, literature on the topic was read to facilitate understanding and interpretation of the text. In the next phase, the analysis utilized interpretive techniques to integrate and juxtapose data with existing theories. Focus was on juxtapose theories and previous research that challenged the narratives, or on finding significant events in the narratives that challenged existing literature (Josephsson & Alsaker, 2015; Mattingly, 1998). Narratives allow for multiple interpretations and by applying an interpretive lens to family experiences, hope can be situated in a context and understood as more than a general desire for something to come true (Bruner, 2004; Mattingly, 2010). To deepen the understanding of experience in everyday life for families where a child with SMA received a new drug treatment, different theories and research of hope were integrated in the analysis. All co-authors critically reviewed all steps in the analysis.

### Trustworthiness and reflexivity

During qualitative data collection and analysis, reflexivity is of utmost importance, because the researcher is a tool in the entire process from data gathering to data analysis (Patton, 2015). To prepare, the first author took methodological courses, practised interviewing technique with children, and performed pilot observations in families. In addition, the last author had extensive experience with narrative interviewing and participant observation as data collection method. Several of the participants asked for information about the first author’s personal and professional background identity at an early stage. Knowledge of some of the researcher’s personal and professional background, such as being a nurse and a parent, might have helped the gaining of trust from the participants. Further, the previous experiences helped the researcher to understand the deeper meanings of the phenomenon that was studied (Carolan, 2003).

During data analysis, it has been of importance for the credibility that the co-authors together covered wide experience with different professions, specific knowledge of the methods used and of SMA. To strengthen the credibility, the authors strive to be aware of own preunderstandings and possible prejudices of the context that was studied. Active discussions occurred within the research group in relation to possible interpretations of data.

### Ethical considerations

Sharing personal experiences through research such as this potentially involves both benefits and risks. Children are considered vulnerable from the perspective that it can be more challenging to make informed decisions in which risks and benefits are weighed. Children are therefore asked to consent to research together with legal guardians that help assure that a child is protected from research that could harm them. Establishing the family-child-researcher relationship involved establishing trust and sharing personal stories; it was thus also relevant to set aside time for achieving closure to the ethnographic process. In this study, researchers and family members reasoned that it was important for the participating families to have their voices represented in research. The risk of time burden or emotional distress among children and families was outweighed by the risks of preventing children and families from research participation, which consequently can contribute to silencing important experiences. In being open about what the research entails and in trusting that children and their families can make their own informed decisions about participation, questions that concern them can contribute to potential benefits of knowledge that can be generated through research. Within this frame, we offer additional descriptions of the process that contribute to exemplify the choices made.

Participant observation can infringe on people’s privacy, something that occurred quite literally in this study, as the researcher became privy to people’s private lives and got intimate information about their habits and routines. The collected data were therefore handled carefully to protect confidentiality. For the purpose of the study, no irrelevant or revealing information about the families were reported. As SMA is rare and the eligible families few, the risk of identification of participants was high. It was therefore necessary to slightly modify the participants’ characteristics or attributes in their environment. The participants were informed of this when they received information about the study.

The researcher (EH) who collected data maintained the ambition to be open and responsive when an informant wanted to share something, but at the same time careful not to intrude, but showing respect for each person’s integrity. Sensitive topics such as vulnerability and death were therefore not taken up by the researcher; however, if the informant introduced a sensitive topic, follow-up questions were asked. As for example, when a sibling told about worries about disease prognosis, open and confirming follow-up questions were asked (e.g., “How does that feel your you?”). The researcher has prior experience in working as a nurse with families in similar situations. This was relevant partly in having strategies for dealing with difficult topics, both for herself and for the families. Although the situation of having a researcher in the home was probably quite unusual for the participants, the mood atmosphere was relaxed. The repeated visits enabled building a shorter relationship between the researcher and the participating families. This might have facilitated the interviews with the children.

In keeping with the declaration of Helsinki, information was given to the participants about the study, that participation was voluntary, that they had the right to withdraw at any time without explanation, and how the collected data would be used. This information was given to the children in an age-appropriate way (Greig et al., 2012). Written informed consent was collected from all participants, and for children younger than 15 years old, written informed consent was also obtained from both parents. Before each visit, the researcher was careful to collect repeated verbal consent to assure to not be overly intrusive. Ethical approval was granted by the regional ethical review board in Stockholm (EPN 2017/985-31/2).

### Description of the families and study context

The results are presented as two family stories where empirical data and discussion are presented together. The two narratives focus on the two boys Alexander and Isaac and their families. As SMA is a rare disease and the eligible families are few, there is a risk of identification of participants. Names and characteristics of the individuals have therefore been modified to protect their privacy and confidentiality.

#### Alexander

Alexander is a thin boy, small for his age with an expressive face. He uses an electric wheelchair which he controls with a joystick for the index finger. When he loose the hand position, he needs help putting the finger in place again. Alexander has difficulties with swallowing, both food and saliva, and due to muscular weakness, his speech is affected. For those who do not know Alexander, it is hard to understand his speech. Despite physical challenges, Alexander expressed confidence and has never expressed any desire to be able to walk or move more. When Alexander was diagnosed with SMA type 1 as a baby, everything became tumultuous for the family. The risk of losing him loomed over his parents and older sibling constantly (Alexander’s younger sibling was born a few years later). Alexander had difficulties with coughing and swallowing and the risk of pneumonia was high. He had multiple life-threatening infections and often received hospital-level care. The infections were frequent, the parents had no time for respite, and the older sibling wondered if Alexander was going to die at hospital. Despite his perceived poor prognosis, Alexander is now attending primary school.

#### Isaac

At the first meeting with Isaac, the first author meets a boy with a smile and intense eyes. Isaac has a cold, as was often the case. Due to a phlegmy cough Isaac has to interrupt his presentation of himself and Isaac’s close relative and both parents has to come to assist with the in-exsufflator cough assist.

Isaac has the diagnose SMA type 2, which keeps him from walking and lifting heavy things, but he can use his hands to handle a pen and paper and hold and operate an iPad. He uses his eyebrows a lot when he talk, raising his eyebrows instead of nodding his head. Sometimes, his head drops forward and he needs help lifting it up again. While Isaac has an active life with friends and activities, he often gets ill and has a school absenteeism of 50% due to infections. Isaac’s infections can appear quickly, and his parents always have to be ready with their tools: the in-exsufflator cough assist, the breathing support and all the different inhalation fluids. Occasionally, the situation gets serious and everything must focus on keeping Isaac breathing.

## Findings and discussion

### Alexander and his family

About one year before the first author’s first visit to Alexander’s family, something happened that meant a big change in the family’s life—Alexander started treatment with nusinersen. Every four months, the family went to a hospital in a larger city for an intrathecal injection with nusinersen given via lumbar puncture in the lower back, during anaesthesia. Alexander found being under anaesthesia scary and felt very cold afterwards. He could not think of many benefits with the treatment when asked, but when his father reminded him of previously needing breathing support in the daytime, Alexander agreed that it is nice to get rid of that.

During the year that had passed since Alexander got his first dose, Alexander had experienced several changes. Unlike before, he now had as much energy as his younger sibling. He attended school full days, and after school, he still had energy to play, often bringing friends home. According to his parents, his muscles had become only a little bit stronger; it was the energy level that made the big difference.

In connection with the treatments at the hospital, Alexander’s family used to combine each trip with something fun. Last year, when Alexander was to receive one of his first doses of nusinersen, they went to the fun fair. At that time, Alexander was easily exhausted and spent most of the time in the fun fair’s rest room. Nearly a year later, they went back to the fun fair and the experience was completely different. Alexander was keen and full of energy all day.

Alexander and his two siblings perceived the disease differently. Alexander did not seem to care or worry about the disease at all. His younger sibling seemed not to worry about Alexander’s disease or that it might threaten his life, maybe since the younger sibling had no experiences from Alexander’s first years of life, when he often was critically ill. By contrast, Alexander’s older sibling grew up with the uncertainty of not knowing if Alexander would survive his next infection. Alexander’s older sibling did sometimes worry about Alexander, due to the knowledge that many with severe SMA pass away at an early age. However, the sibling tried not to think about that, and focus on Alexander now living in health.

The only thing that has been, that I think about a bit, is if he’ll get to be like a middle-aged person and die when he’s, like, 80 years old, or if it’ll be at 15 or something. That’s basically the only thing. But it’s hard to say. But right now, I hope, and it looks like he’ll lead a normal life, just that he has a disease and can’t move in the same way.

Alexander’s older sibling

Parents of children with neuromuscular diseases can vacillate between hope for future treatments, avoidance of emotionally difficult aspects of the disease, and living in the present (Erby et al., 2006). This applied also to Alexander’s sibling, who conveyed a sense of a good everyday life and a positive outlook on the future, while at the same time having underlying uncertainties and doubts. Alexander’s father said that he, due to Alexander’s diagnosis, had learned not to hope for too much. In contrast to other parents he had seen, who placed much hope in potential therapies and became disappointed and bitter when they did not work, he wanted to focus on appreciating his time with Alexander. As a result of Alexander’s diagnosis and all the uncertainty it had brought into the father’s life, he said that he had learned to appreciate nice moments in everyday life, without thinking about the future. This life skill that he had gained was something that he cared about. On the back of this, Alexander’s father stated that he did not have big expectations or hopes regarding the new therapy.

So, I don’t think that like … In two years, he’ll be able to do this or that – we’ll just take it … Right now, things are good, so that’s good. And it’s nice as heck to have that insight.

Alexander’s father

In addition, Alexander’s father said that he, rather than hoping Alexander’s muscles would get stronger, was hoping for improved quality of life, and he felt that they had already achieved this. Hope can take on many different forms; for instance, it can be related to strategies for coping with adversities (Folkman, 2010) and can be viewed as a dynamic work a person with life-threating disease can perform (Schaufel et al., 2011). This way of using hope as coping strategy can be trained (Mattingly, 2010). Alexander’s father had developed his own strategy, which involved appreciating the present and benefits already achieved. A new hope of another way of life, with possibilities to plan for future, would challenge this strategy of acceptance, and a change, even for the better, would require reassessment, something that would probably necessitate both emotional and cognitive efforts.

From Alexander’s mother’s perspective, the new medicine had allowed her to partially relax. For the first time, she dared to feel hope for the future—hope that there was a future for Alexander. Before the treatment with nusinersen began, Alexander’s mother saw how Alexander got increasingly fatigued as the years passed. Also before he was always happy, but forced to spend more of his time resting. Now, suddenly, Alexander did not want to rest anymore. However, despite the new calmer situation that the medicine brought to the family’s life, new challenges had appeared in Alexander’s mother’s everyday life. She told that she was not satisfied with her life, as she was herself experiencing a personal life crisis. Some years ago, Alexander’s mother came to a point where she concluded that she had to quit working. She could not lead a balanced life, combining work and parenting. Now, life at home had become agonizing. She described the walls of their house almost like a prison.

I walk around here at home and clean and do the laundry and cook and … I don’t really do anything else. I don’t meet many friends; I never get up to anything. I and (Alex’s father) don’t have much time together. Uhm … I don’t go to any Christmas parties, like everyone else. So, it’s gotten a bit … I feel like I’m almost becoming bitter, and feel that I need to make an effort so that I get more of a social life.

Alexander’s mother

Alexander’s mother felt self-doubt; she wanted to get back to work, but was unsure about what she could manage, and whether anyone would want to employ her. Feelings of social isolation and problems with combining care of a sick child with employment have previously been described among parents of children with disabilities (Brown & Clark, 2017; Heiman, 2002; Redquest et al., 2015). In Alexander’s mother’s case, the feelings of isolation had grown as Alexander’s health had become more stable.

Crises and persistent life challenges for one family member have an impact on the whole family, and important processes for individual family members affect relationships in the entire family unit. In this case, the new relief and hope affected Alexander’s mother in another direction (Wright & Leahey, 2012). This can be understood as Alexander’s family constantly being in movement between change and balance, stagnation and dynamics, security and uncertainty—a family in a crisis searches for stability, while a family that perceives stagnation seeks change (Walsh, 2016; Wright & Leahey, 2012).

### Isaac and his family

Over the course of the winter during which this study was conducted, something occurred that had never happened to Isaac before; everyone in his family fell ill with a fever and a cold—except Isaac. Isaac giggled when he told about this. Since Isaac had started treatment with nusinersen a few months earlier, he could cope better with infections. Isaac said that he was treated with nusinersen to make him stronger and able to walk. He felt, after just a few injections, that he had become a little stronger, for example, he could raise his arm more when lying in bed, and had more mobility in his legs. Isaac was goal-oriented and confident that he would be able to walk someday. In the future, he thought he would work as a veterinarian—not sitting in a wheelchair.

Like Isaac, his mother was convinced that Isaac would someday be able to walk. She was not certain that nusinersen on its own would make him walk, but had faith in other new therapies as well.

There’s a lot of research on this and that they’ve found different ways to do it. […] So, you absolutely have to believe that. At some point … they’ll find something. And then the people making the decisions have to be quick.

Isaac’s mother

However, hope for treatment can also provide a tool for parents to be supportive of their child’s own dreams, even if the parents do not believe in them (Erby et al., 2006). This applies to Isaac’s father, who was not sure that Isaac would be able to walk, but did not want to take Isaac’s hope away. He thought it *could* be possible, but added that both he and Isaac knew there was a long way to go, given the limited muscle strength that Isaac had. Isaac’s close relative was more cautious in her hopes regarding the treatment. She did not dare be filled with hope, worried that having high expectations would lead to disappointment. Here it is relevant to note that since nusinersen is new, there are no documented long-term prognoses or statistical data on the treatment. It is therefore impossible to say if the hope of Isaac walking was realistic. On the other hand, statistics play a minor role in families’ ability to feel hope. Clinicians and families can use contrasting languages to consider hope and make differing assessments about what should realistically be hoped for (Mattingly, 2010). Mattingly (2010) has earlier described how a clinician’s language of statistical probabilities can depart sharply from the spiritual discourse of hope that is common in many families. This could mean that Isaac and his mother do not reject the medical discourse of probabilities; rather, they may feel it presents only part of the picture. Further, literature indicates that people with unrealistic optimism about their ability to manage traumatic events may have an effective coping style (Taylor & Armor, 1996), and hope for cure is not necessarily a problem, despite poor prognosis (Robinson, 2012; Van der Geest et al., 2015). Still, it is important to note that hope based on false information is never recommended (Groopman, 2004; Snyder, 2002).

Isaac’s sibling did not see the disease as a threat to Isaac’s life, but could feel sorry for Isaac sometimes, when he was prevented from participating in activities due to his SMA. The sibling wished that everything could be adapted for everyone, including persons with disabilities. The narrative of the sibling’s concern over equal rights to a normal life is noteworthy, as it was identified as representing a hope beyond that of survival but for continuing life with possibilities, dignity, and good quality. Bérubé (1998) has reflected on society’s responsibility for children with disabilities, and argues that when society, as a result of antibiotics, modern surgery, drug treatment, and early intervention programmes, saves children with severe disabilities, it also has a responsibility to enable those children to flourish and lead normal lives. This might be relevant, as children with SMA undergoing treatment with nusinersen are expected to live longer, possibly with continuing physical impairment (Pane et al., 2018; Pechmann et al., 2018).

Isaac explained that he did not like to exercise, but that he did it anyway to activate his body and muscles. The hope for treatment results increased Isaac’s motivation for exercise and treatment compliance, something that is important for better outcome of nusinersen. When seen from a perspective of medical management, hope can be a predictor of medication compliance among children with a chronic disease (Berg et al., 2007). Seen from Isaac’s perspective, hope was a personal impetus to achieve things that he wanted to.

It can be difficult to talk about the concept of hope in families’ everyday lives with severe disease without also reflecting on resilience. The concept of family resilience refers to family capabilities to regain psychological and functional integrity after adversity. Family resilience is a process of adaptation and can be affected and changed, both at an individual level and within a family system (Oh & Chang, 2014). Isaac and his family were perceived as resilient and had the ability to see opportunities rather than obstacles. They conveyed strategies for dealing with everyday life that were characterized by family resilience, e.g., conveying a positive picture of their life with few adversities, a tendency to distinguish what they could change from what they could not, and sensitivity in finding and being grateful for the small things in life (Oh & Chang, 2014). There is an interrelationship between hope and resilience, but the association between the two concepts is not clear in literature. It is difficult to determine which of them contributes to the other. Several studies show that hope is a protective factor contributing to resilience (Earvolino‐Ramirez, 2007; Gillespie et al., 2007), while others emphasize that persons who have resilient characteristics also have the ability to feel hope (Snyder, 2000).

Hope is often described as dynamic and capable of existing at multiple levels and taking different pathways (Bally et al., 2014; Duggleby et al., 2010; Leite et al., 2019; Mattingly, 2010), which can explain how it could present so differently for the families in this study.

## Methodological considerations

The use of narratives situated in everyday life provides knowledge about how hope is enacted in family life and a family’s co-constructed stories. Or, as Mattingly puts it: “to insist that if hope is to be discovered in all its vagaries, vulnerabilities, and paradoxes, one must look to personal and family life” (Mattingly, 2010, s. 233).

There is more than one way to construct and interpret a text (Polkinghorne, 2007; Ricoeur, 2008). The empirical data in this study, generated with two families, should be viewed as co-constructions between the participants and the researchers, where the reconstructed narrative and interpretations were created by the researchers (Mattingly, 1998; Polkinghorne, 2007). This approach is in line with the social constructivist epistemology and view of knowledge as constructed between people (Burr, 2015).

Children are often compliant in their communication, and might answer what they think the researcher wants to hear. The interviewer was aware of this and actively tried to create an inviting context with open questions, to help the children feel safe and share experiences from their perspective to the greatest extent possible (Nicol et al., 2017).

To enable the reader to follow the research process and judge the transferability, the researchers have strived to be conscientious in method descriptions, interpretations, and presentation of data. The descriptions of the families and their dynamics, together with the theoretical resources described, provide readers with information to assess the transferability and plausibility of the interpretation offered (Polkinghorne, 2007). The findings may be of value also in relation to families with other severe illnesses other than SMA.

Both participating families were possessing resilience with open communication within the family units. It should be kept in mind that families that are not communicative would probably not agree to participate in this type of study. It is emphasized that this study does not focus on, or draw any conclusions about, the medical effects of nusinersen.

## Conclusions

Hope can take different shapes and be a source of strength, which is why it is of value to reflect on how families nurture hope. This study showed that the change in energy and activity levels among the children with SMA, together with family hope for future possibilities, could be expressed differently by different family members. Some family members may be convinced that the treatment will remove all symptoms of the disease, while some do not dare to hope for too much, and for others the relief brought by the hope, may give rise to new challenges—all these completely different approaches can be seen as different negotiations with everyday hope for a good future.

Narrative allows for a writing and rewriting of the family story based on new experiences or changing circumstances. The turning point of a new drug had in these cases shifted from focusing on a struggle with day-to-day challenges and a fight to stay alive, to focusing on a promising future. The hope was negotiated and struggled with in different ways by different family members, but contributed to each family member’s own way of dealing with the disease and their outlook on the future. Our findings show how hope in everyday life, whether realistic or not, has the potential to strengthen children and parents in their way of looking at the disease and future.
